# Supplementary material for: Foaming and Physicochemical Properties of Commercial Protein Ingredients Used for Infant Formula Formulation
Source: Foods. 2022 Nov 18;11(22):3710. doi: 10.3390/foods11223710 (PMC9689407; doi:10.3390/foods11223710)
Supplement: Supplementary file 1 [file foods-11-03710-s001.zip › foods-1982311-supplementary.pdf]

Table S1 The chemical composition of D70, D90, WPC, and SMP.

Sample		Source	Manufacturers	Protein (%)	Lactose (%)	Fat (%)	Moisture (%)	Ash (%)
D70	1	Ireland	Dairygold Food Ingredients Co., Ltd	13.69	81.75	1.18	0.99	2.17
	2	Finland	Valio Co., Ltd	13.00	81.90	0.90	1.40	2.80
	3	Germany	Alpavit Co., Ltd	12.50	83.20	0.70	1.10	2.60
	4	France	Euroserum Co., Ltd	12.60	82.10	0.90	1.80	2.70
	5	France	Lactalis Co., Ltd.	12.66	82.20	1.20	1.60	2.50
D90	1	France	Lactalis Co., Ltd.	13.59	79.00	1.20	0.85	0.54
	2	Germany	Alpavit Co., Ltd.	12.80	84.47	0.84	1.37	0.70
	3	Finland	Valio Co., Ltd.	12.90	84.10	0.90	1.20	0.70
	4	Netherlands	Frieslandcampina Co., Ltd.	13.50	83.00	0.30	2.10	0.80
	5	Germany	Hochwald Foods Whey Ingredients Co., Ltd	12.87	80.00	0.60	1.19	0.84
WPC	1	Germany	LIMEI Co., Ltd.	80.80	6.80	4.50	4.30	3.60
	2	America	Hilmar Ingredients Co., Ltd.	78.51	7.02	4.53	4.31	4.93
	3	Germany	Sachesenmilch Co., Ltd.	77.68	7.05	4.68	4.43	3.92
	4	Germany	Wheyco Co., Ltd.	77.77	7.09	5.67	4.81	4.66
	5	Germany	Hochwald Foods Whey Ingredients Co., Ltd.	76.40	6.90	6.60	4.60	2.50
SMP	1	Australia	Union Dairy Co., Ltd.	34.70	53.46	0.80	4.17	6.87
	2	Germany	Molkerei Ammerland Co., Ltd.	32.90	55.40	0.54	2.71	7.96
	3	Finland	Valio Co., Ltd.	38.00	50.00	0.54	3.80	7.79
	4	Ireland	Kerry Co., Ltd.	34.97	54.20	0.70	3.20	7.60
	5	Ireland	Dairygold Food Ingredients Co., Ltd.	35.00	53.80	0.90	3.20	7.10

Table S2. Foaming and physicochemical properties of D70, D90, WPC, and SMP.

	D70	D90	WPC	SMP
Foaming capacity (%)	80.3 ± 9.7[ab]	78.8 ± 8.6[ab]	74.1 ± 9.7[b]	90.7 ± 9.7[a]
Foam stability (%)	37.5 ± 18.7[b]	31.6 ± 13.0[bc]	16.5 ± 4.3[c]	58.2 ± 5.5[a]
Particle size (nm)	261.4 ± 33.9[ab]	227.7 ± 23.5[b]	280.5 ± 47.9[a]	223.6 ± 15.5[b]
Zeta potential (mV)	-22.3 ± 1.9[b]	-25.3 ± 4.2[b]	-23.7 ± 4.0[b]	-17.6± 0.9[a]
Turbidity	0.30 ± 0.09[b]	0.20 ± 0.01[b]	0.27 ± 0.09[b]	0.73 ± 0.13[a]
Solubility (%)	93.7 ± 3.8[a]	88.5 ± 6.9[a]	86.4 ± 6.6[a]	65.5 ± 6.1[b]
Surface hydrophobicity	5014.6 ± 556.6[a]	4667.6 ± 702.7[a]	3138.4 ± 186.4[b]	2999.5 ± 245.6[b]
Free sulfhydryl content (μmol/g)	15.9 ± 3.2[a]	13.4 ± 3.8[a]	13.6 ± 4.0[a]	5.1 ± 0.5[b]
α-helix (%)	15.9 ± 0.6[ab]	16.2 ± 0.8[b]	15.6 ± 1.6[a]	14.9 ± 0.4[c]
β-sheet (%)	28.2 ± 0.6[b]	27.9 ± 0.8[b]	29.2 ± 1.4[a]	25.6 ± 0.6[c]
β-turn (%)	23.5 ± 0.4[b]	23.6 ± 0.3[b]	22.9 ± 0.4[c]	24.9 ± 0.2[a]
Random coil (%)	32.3 ± 0.4[b]	32.3 ± 0.3[b]	32.3 ± 0.1[b]	36.4± 0.3[a]

Different letters (a-c) indicate significant differences at $p < 0.05$.
